# A Critical Overview of Systematic Reviews and Meta-Analyses of Acupuncture for Female Stress Urinary Incontinence

**DOI:** 10.1155/2022/5887862

**Published:** 2022-05-17

**Authors:** Hongshuo Shi, Leizuo Zhao, Lirong Cui, Zicheng Wang, Dan Wang, Pulin Liu, Guomin Si, Dong Guo, Wenbin Liu

**Affiliations:** ^1^College of Traditional Chinese Medicine, Shandong University of Traditional Chinese Medicine, Jinan, China; ^2^Department of Urology, Dongying People's Hospital, Dongying, China; ^3^Shandong College of Traditional Chinese Medicine, Yantai, China; ^4^Department of Urology, Provincial Hospital Affiliated to Shandong First Medical University, Jinan, China; ^5^Department of Traditional Chinese Medicine, Provincial Hospital Affiliated to Shandong First Medical University, Jinan, China; ^6^Center for Faculty Development, Shandong University of Traditional Chinese Medicine, Jinan, China; ^7^The Second Affiliated Hospital of Shandong University of Traditional Chinese Medicine, Jinan, China

## Abstract

**Objectives:**

As a urinary dysfunction disorder, stress urinary incontinence (SUI) is more common in women than in men. Acupuncture, a traditional minimally invasive technique, has potential efficacy in the treatment of SUI. The purpose of this overview is to critically assess the available evidence on acupuncture for the treatment of SUI in women.

**Methods:**

Two researchers searched seven databases for systematic reviews (SRs)/meta-analyses (MAs) of randomized controlled trials (RCTs) on acupuncture for SUI. Two researchers assessed the included SRs/MAs using the Assessment of Multiple Systematic Reviews 2 (AMSTAR-2), the Risk of Bias in Systematic (ROBIS) scale, the list of Preferred Reporting Items for Systematic Reviews and Meta-Analysis (PRISMA), and the Grading of Recommendations Assessment, Development, and Evaluation (GRADE) system.

**Results:**

Eight published SRs/MAs were included in our overview. According to the results of the AMSTAR-2 assessment, all SRs/MAs were of very low quality. According to the ROBIS evaluation results, no SR/MA was assessed as low risk of bias. According to the results of the PRISMA checklist assessment, no SR/MA was fully reported on the checklist. According to GRADE, a total of 27 outcomes extracted from the included SRs/MAs were evaluated, and only 1 was rated as high quality.

**Conclusions:**

Acupuncture may be an effective and safe complementary treatment for SUI in women. However, further standard and comprehensive SRs/MAs and RCTs are needed to provide an evidence-based medical rationale for this.

## 1. Introduction

As a form of dysfunction disorder, stress urinary incontinence (SUI) is the most common type of urinary incontinence. It is defined as the involuntary flow of urine due to physical exertion or effort, coughing, or sneezing [1]. SUI has a prevalence of up to 49% in physically active women and up to 15% in women aged 30–60 [2]. SUI can cause psychological burden, affect relationships, decrease physical productivity, and reduce a woman's quality of life [3], and its harm is even greater than that of major chronic diseases such as diabetes, hyperlipidemia, and chronic kidney diseases [4]. However, more than 80 percent of women received no treatment at all [5].

The main treatment modalities for SUI include lifestyle interventions, electrical stimulation, pelvic floor muscle training (PFMT), medication, and surgery [6]. The American Urological Association (AUA) currently recommends conservative treatment, such as PFMT, for patients with mild and moderate SUI [7]. However, this method has shortcomings such as poor compliance and difficulty in mastering training skills. Surgical treatment is effective in patients with severe SUI, but it can cause potential complications, including pain, infection, and dysuria [8]. Therefore, there remains an urgent need for an effective and safe complementary treatment for SUI.

As a minimally invasive treatment method, acupuncture has a history of more than 2,500 years in China and is gaining more and more international attention in the field of healthcare [9]. With its unique advantages, acupuncture plays an irreplaceable role in the treatment of SUI. With the extensive use of acupuncture in the treatment of SUI, related systematic reviews (SRs) and meta-analyses (MAs) have also been published. Since the evidence provided by these SRs/MAs for acupuncture for SUI in women is sometimes inconsistent and varies in quality, a reevaluation is needed. An overview of SRs/MAs is a new method to comprehensively assess the methodological quality and certainty of quality across multiple SRs/MAs. Therefore, our research aimed to critically evaluate the quality of SRs/MAs regarding the acupuncture for female SUI through a comprehensive overview.

## 2. Materials and Methods

The methodology of this study follows the Cochrane manual, as well as the study methods of some high-quality SRs/MAs overviews [10–12].

### 2.1. Inclusion and Exclusion Criteria

The criteria for inclusion of SRs/MAs in this overview are as follows: (1) Study design: This overview includes SRs/MAs of randomized controlled trials (RCTs) of the acupuncture on SUI; (2) Type of participants: Female subjects diagnosed with SUI based on any authoritative national or international diagnostic criteria regardless of race, age, gender, time of onset, and source of cases; (3) Intervention: The control group received the following treatments: Conventional medication (CM), rehabilitation training (RT), sham acupuncture (SA), and placebo. The intervention group received acupuncture treatment, including plum blossom acupuncture, fire acupuncture, electro-acupuncture, body acupuncture, manual acupuncture, warm acupuncture, or acupuncture therapy in combination with the treatments received by the control group; (4) Outcome indicators: Outcomes assessed in this overview include: Effective rate, 1-hour pad test, international consultation on incontinence questionnaire short form (ICIQ-SF) score, visual analog scale (VAS) score, and adverse reactions.

The criteria for exclusion of SRs/MAs in this overview are as follows: (1) Animal studies; (2) Network MAs, research protocols, narrative reviews, overviews, dissertation, and conference abstracts.

### 2.2. Search Strategy

Literatures were retrieved from PubMed, Cochrane Library, EMBASE, Chongqing VIP, Wanfang Database, CNKI, and SinoMed on 1 January 2022. We adopted a search strategy combining keywords with free words, and the keywords include acupuncture, urinary incontinence, systematic review, and meta-analysis. The literature search strategy (shown in Table 1) of the PubMed database was reasonably tuned for each database. We also reviewed the references of the all retrieved literature to avoid missing topic-related SRs/MAs.

### 2.3. Literature Screening and Data Extraction

The literature screening (HS-S and LZ-Z) and information extraction (WB-L and ZC-W) were performed independently by two researchers. We firstly input the retrieved documents into Endnote X9 document management software, and then removed the duplicates. The literatures that potentially met the inclusion and exclusion criteria were then obtained by reading the titles and abstracts. Eventually, we finalized the included SRs/MAs by reading the full text. A standardized data extraction form was adopted to extract relevant information for the inclusion of SRs/MAs. The following information was extracted from each SR/MA: First author, year of publication, author nationality, number of RCTs included, sample size, intervention group measures, control group measures, tools used to assess the risk of bias, and main findings.

### 2.4. Quality Assessment for Inclusion in SRs/MAs

Two researchers (PL-L and D-W) independently assessed the methodological quality and certainty of quality of the included SRs/MAs.

#### 2.4.1. Assessment of Methodological Quality

The methodological quality of the included SRs/MAs was assessed by the Assessment System for Evaluating Methodological Quality 2 (AMSTAR-2) [13]. Seven (2, 4, 7, 9, 11, 13, and 15) of the 16 items in the tool are critical areas.

#### 2.4.2. Assessment of Risk of Bias

The Risk of Bias in Systematic Review (ROBIS) [14] scale was used in this overview to evaluate the risk of bias in the inclusion of SRs/MAs and the evaluation was carried out in three stages.

#### 2.4.3. Assessment of Reporting Quality

The quality of each SR/MA report of the included SRs/MAs was evaluated by the list of PRISMA [15] which consists of 27 items focusing on the reporting methods and results that were incorporated into SRs/MAs.

#### 2.4.4. Assessment of Certainty of Quality

The certainty of quality for each SR/MA outcome was evaluated by The Grading of Recommendations Assessment, Development, and Evaluation (GRADE) [16], and five aspects will lead to the degradation of certainty of quality, including limitations, inconsistencies, indirectness, imprecision, and publication bias.

## 3. Results

### 3.1. Literature Search and Screening Results

**Figure 1:**
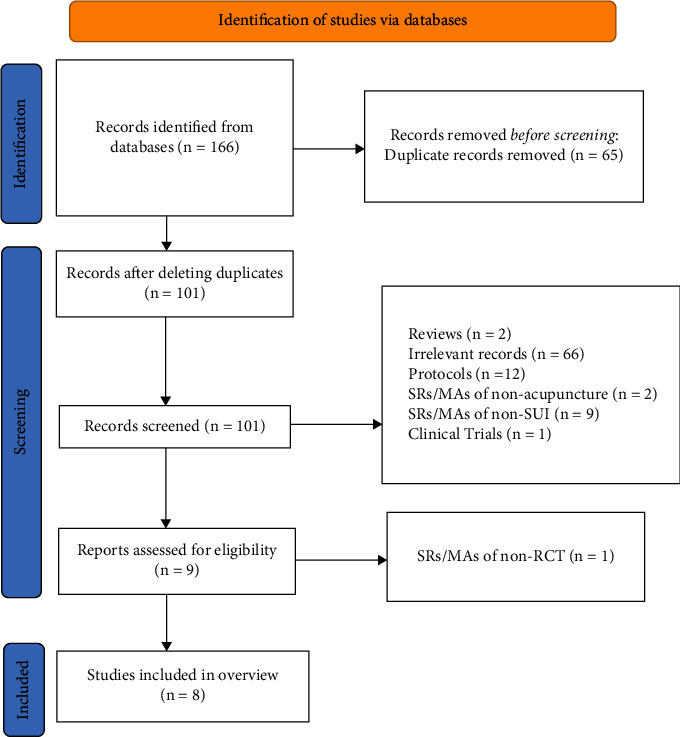
The flowchart of the screening process.

A total of 166 publications were retrieved from seven electronic databases. Nine publications were retrieved for full-text evaluation after the duplicates removal and title/abstract screening. One paper [17] was excluded because it didn't focus on RCTs, and the remaining 8 SRs/MAs [18–25] were included in this overview. The flowchart of the screening process is shown in Figure 1. The exclusion list for the literature is in Table 2.

### 3.2. Inclusion of Characteristics of SRs/MAs

The characteristics of the 8 SRs/MAs included in our final evaluation were summarized, as shown in Table 3. These SRs/MAs were published between 2014 and 2021, with 6 [18–23] published after 2017. Three of the SRs/MAs [18–20] were in English, and the remaining five [21–25] were in Chinese. The number of RCTs included in the SRs/MAs ranged from 9 to 15, and the total number of subjects included in each SR/MA ranged from 579 to 1,577. The interventions for the control group were CM, SA, RT, and placebo, and the treatments for the intervention group were electroacupuncture (EA) and manual acupuncture (MA) or EA or MA in combination with the treatments received by the control group. In terms of the quality assessment for inclusion in RCTs, the Cochrane criteria was used for four SRs/MAs [18–20, 23] and the Jadad scale was used for four SRs/MAs [21, 22, 24, 25].

### 3.3. Results on SRs/MAs Quality Assessment

#### 3.3.1. Results of the Methodological Quality

Regarding the methodological quality of the included SRs/MAs, all were considered to be of very low quality because more than one key item was missing from the SRs/MAs included in the quality assessment. Methodological quality limitations come from the following items: Items 2 (Only 2 SRs/MAs [19, 20] registered the research protocol), Item 4 (None of the SR/MA performed a comprehensive literature search), Item 7 (None of the SR/MA provided studies excluded from the list), and Item 10 (None of the SR/MA reported the funding of RCTs included in SRs/MAs). The evaluation details of the included SRs/MAs on the AMSTAR-2 are shown in Table 4.

#### 3.3.2. Results of the Risk of Bias Assessment

Regarding the results of the ROBIS assessment, Phase 1 assessed the relevance of the study topic and Domain 1, with all SRs/MAs rated as low risk of bias in both items. Domain 2 assessed the identification and selection of studies, and none of the SR/MAs had a low risk of bias. In Domain 3, 6 SRs/MAs [18–22, 25] were rated as low risk of bias. Domain 4 assessed the synthesis and findings, and only 2 SRs/MAs [19, 23] were rated as low risk of bias. Phase 3 considered the overall risk of bias in the reviews, and none of the SR/MA had a low risk of bias. The evaluation details of the included SRs/MAs on the ROBIS scale are shown in Table 5.

#### 3.3.3. Report Quality of the Included SRs/MAs

The results of the PRISMA inventory evaluation were shown in Table 6. 22 out of 27 items have a “yes OR partially yes” response rate of more than 60%, and this shows that the report is relatively complete. However, there are some reporting deficiencies in other items. The reports of Items 7 (search strategy), Item 15 (certainty assessment), Item 22 (competing interests), Item 23 (certainty of evidence), and Item 24 (registration and protocol) are incomplete (the “yes OR partially yes” response rate is less than 50%).

#### 3.3.4. Results of the Certainty of Quality

The 8 SRs/MAs included 27 outcomes related to the effectiveness of acupuncture for SUI. For all the outcome indicators, 1 was rated as high quality, 10 moderate, 6 low and 10 very low by means of the GRADE evaluation. Publication bias (*n* = 24) was the most common downgrading factor, followed by risk of bias (*n* = 13), inconsistency (*n* = 13), imprecision (*n* = 9), and indirectness (*n* = 0). GRADE specific assessment details are shown in Table 7.

### 3.4. Description of Efficacy and Safety

Details of outcomes included in SRs/MAs are shown in Table 8, and 2 SRs/MAs [19, 20] provide narrative reviews that regard acupuncture as a safe treatment option. It can be seen that acupuncture is effective and safe for the treatment of female SUI.

## 4. Discussion

SUI can severely impair a patient's ability to perform daily activities, leading to embarrassment, insomnia, and social isolation [26], and acupuncture is a minimally invasive technique with the potential treatment of SUI. This research aimed to systematically and comprehensively collate, evaluate and summarize the published evidence on acupuncture for SUI in recent years.

### 4.1. Summary of the Main Findings

This overview incorporated 8 SRs/MAs on acupuncture for SUI. These publications were based on the RCT and were published from 2014 to 2021. Six (6/8, 75%) SRs/MAs were published in the last five years, indicating that acupuncture had received increasing attention as an important intervention modality for SUI in women.

Based on the results of the AMSTAR-2, ROBIS, and PRISMA evaluation in this overview, the methodological and reporting quality of the SRs/MAs were unsatisfactory. Only two SRs/MAs contained initial research protocol registrations, the lack of which could lead to non-standardization of the research process, increase the risk of bias and impact the rigor and credibility of the final SRs/MAs results. All of the included SRs/MAs lacked a search of the gray literature, which made it difficult to ensure the comprehensiveness of the literature search and tended to generate publication bias. None of the SR/MA provided a complete list of exclusions for each study, which may affect the reliability of the results and assessment of publication bias. The provision of a list of exclusion researches can be a stronger demonstration of the rigor of the literature screening process. No SR/MA reporting was included in the RCT's funding resources, which may increase the bias in clinical trials as the results of corporate-funded studies may be biased in favor of the funder. None of the SR/MA provides comprehensive search strategies, which reduced the reproducibility and credibility of the study. In addition, the lack of reporting of conflicts of interest also potentially affected the credibility of the article.

Based on the GRADE assessment, publication bias was deemed as the most significant downgrading factor. Further analysis revealed a risk of publication bias for the outcome indicators included in the SRs/MAs, which may be related to incomplete searches and the insufficient number of RCTs included in the relevant outcomes. In addition, other reasons for the downgrading risk of bias included: Most of these RCTs mentioned randomization without giving the randomization method; most didn't conceal allocation; and most didn't use blinding method or just used single blinding. Declining certainty of quality due to inconsistency may stem from substantial clinical and methodological differences in the included RCTs, which could be avoided by standardizing the inclusion and exclusion criteria as well as the literature screening process.

Descriptive analysis suggested that acupuncture was an effective treatment for SUI in women with a high safety profile. However, due to the low methodological quality and certainty of quality of the included studies, these findings may deviate from the actual results. Therefore, caution should be exercised when recommending acupuncture as a complementary intervention for SUI in women.

### 4.2. Implications for Practice and Research

Featuring unique advantages, acupuncture therapy plays an integral role in the treatment of urinary incontinence. Acupuncture works by repeatedly stimulating the points within the body by needling the control points of the bladder and sphincter muscles, thus effectively repairing and improving the body's various control functions [27].

This paper gave a comprehensive assessment of all aspects of the included SRs/MAs using AMSTAR-2, PRISMA, ROBIS, and GRADE, and the methodological quality and certainty of quality were found unsatisfactory. As implied, there is still considerable scope for addressing the quality issues in the process of conducting SRs/MAs. When selecting topics for SRs/MAs, investigators should register or publish study protocols in advance to minimize the risk of bias and ensure the standardization of SRs/MAs. The search for gray literature, complete search strategy and the list of excluded literature need to be supplemented in SRs/MAs, which can reduce publication bias and improve the certainty of quality. A list of funding for RCTs and declarations of conflicts of interest need to be provided to increase the credibility of SRs/MAs. In addition to this, the specific nature of acupuncture treatment makes it difficult to perform a blinded acupuncture-related RCT. However, patients, care providers, and outcome evaluators should be blinded whenever possible to minimize the risk of bias. A well-designed and rigorously executed RCT is believed to be the gold standard for evaluating interventions to minimize or avoid bias [28]. Acupuncture has its origins in TCM theory, and there exist a diverse and individualized selection of acupuncture points, and future research protocols on acupuncture should be fully standardized to improve the quality of research. In addition, the currently published SRs/MAs neglect the assessment of indicators related to urodynamic indices, and it is expected that future studies should help us to better understand the potential mechanism of acupuncture for SUI by increasing the assessment of related indicators.

### 4.3. Strength and Limitations

Our overview is the first to use AMSTAR2, ROBIS, PRISMA, and GRADE to evaluate SRs/MAs regarding acupuncture for the treatment of female SUI. The evaluation process revealed clear limitations of the current relevant SRs/MAs and RCTs, which may help boost the quality of future clinical studies. However, the overview may have some limitations due to the subjectivity of the evaluation. Although our evaluation had been assessed and reviewed by two independent reviewers, different reviewers may have their own judgments about each factor, so the results may vary. In addition, for different SRs/MAs included in this overview, the definition of effective rate is not mentioned.

## 5. Conclusion

In conclusion, acupuncture is a beneficial and safe way to treat SUI in women. However, due to the generally low methodological quality and certainty of quality in the included SRs/MAs, clinicians should approach these findings with caution in their practice.

## Figures and Tables

**Table 1:** Search strategy for the PubMed database.

Query	Search terms
^#^1	“Acupuncture” [mesh]
^#^2	“Pharmacopuncture” OR “acupuncture” OR “plum blossom needle” OR “fire needling” OR “warm needling” OR “electroacupuncture”
^#^3	^#^1 OR ^#^2
^#^4	“Urinary Incontinence”[Mesh]
^#^5	“Incontinence, urinary”, “urinary incontinence”
^#^6	^#^4 OR ^#^5
^#^7	“Urinary incontinence, stress”[mesh]
^#^8	“Urinary stress incontinence” or “incontinence, urinary stress” or “stress incontinence, urinary” or “stress urinary incontinence”
^#^9	^#^7 OR ^#^8
^#^10	^#^6 OR ^#^9
^#^11	Meta-analysis as topic [mesh]
^#^12	“Systematic review” OR “meta-analysis” OR “meta analysis” OR “meta-analyses” OR “review, systematic” OR “systematic reviews”
^#^13	^#^12 OR ^#^13
^#^14	^#^3 AND ^#^10 AND ^#^13

**Table 2:** 

Report excluded	Reason for Exclusion
Cheng, P., Chi, Z., Xiao, Y., Xie, W., Zhu, D., Yu, T., Jiao, L. (2020). The acupuncture-related therapy for post-stroke urinary incontinence: A protocol for systematic review and network meta-analysis. Medicine (Baltimore), 99(44), e22865. doi:10.1097/md.0000000000022865	Research Protocol
Huang, W., Li, X., Wang, Y., Yan, X., & Wu, S. (2017). Electroacupuncture for women with stress urinary incontinence: Protocol for a systematic review and meta-analysis. Medicine (Baltimore), 96(49), e9110. doi:10.1097/md.0000000000009110	Research Protocol
Lin, Q., Ren, Y., Chen, K., Duan, H., Chen, M., & Liu, C. (2021). Efficacy and safety of heat-sensitive moxibustion in the treatment of neurogenic bladder after spinal cord injury: A protocol for systematic review and meta-analysis. Medicine (Baltimore), 100(24), e26424. doi:10.1097/md.0000000000026424	Research Protocol
Mo, Q., Wang, Y., Ye, Y., Yu, J., & Liu, Z. (2015). Acupuncture for adults with overactive bladder: a systematic review protocol. BMJ Open, 5(1), e006756. doi:10.1136/bmjopen-2014-006756	Research Protocol
Su, T., Zhou, J., Liu, Z., Chen, Y., Zhang, W., Chu, H., Liu, B. (2015). The efficacy of electroacupuncture for the treatment of simple female stress urinary incontinence - comparison with pelvic floor muscle training: study protocol for a multicenter randomized controlled trial. Trials, 16, 45. doi:10.1186/s13063-015-0560-1	Research Protocol
Sun, Z., Yu, N., Yue, J., & Zhang, Q. (2016). Acupuncture for urinary incontinence after stroke: a protocol for systematic review. BMJ Open, 6(2), e008062. doi:10.1136/bmjopen-2015-008062	Research Protocol
Wang, P., Shi, J., Zhao, L., Li, M., Jiao, J., Li, L., Zhang, S. (2020). The efficacy and safety of electroacupuncture against urinary incontinence after stroke: A protocol for systematic review and meta analysis. Medicine (Baltimore), 99(38), e22275. doi:10.1097/md.0000000000022275	Research Protocol
Wang, T. S., Wang, Z. M., Zhao, Y., Tang, Z. C., Song, W. D., & Wang, G. K. (2020). Effectiveness of electroacupuncture (EA) for the treatment of urinary incontinence (UI) in patients with spinal cord injury (SCI): A protocol of systematic review of randomized controlled trials. Medicine (Baltimore), 99(30), e21077. doi:10.1097/md.0000000000021077	Research Protocol
Wang, Y., Li, H., Wang, J., Hao, Q., Tu, Y., Chen, Y., Zhu, T. (2020). A network meta-analysis protocol of conservative interventions for urinary incontinence in postpartum women. Medicine (Baltimore), 99(33), e21772. doi:10.1097/md.0000000000021772	Research Protocol
Yang, J., Cheng, Y., Zhao, L., Chen, J., Zheng, Q., Guo, Y., & Liang, F. (2020). Acupuncture and related therapies for stress urinary incontinence: A protocol for systematic review and network meta-analysis. Medicine (Baltimore), 99(28), e21033. doi:10.1097/md.0000000000021033	Research Protocol
Zhong, D., Tang, W., Geng, D., & He, C. (2019). Efficacy and safety of acupuncture therapy for urinary incontinence in women: A systematic review and meta- analysis. Medicine (Baltimore), 98(40), e17320. doi:10.1097/md.000000000001732	Research Protocol
Zhu, Z., Zhuo, Y., Jin, H., Wu, B., & Li, Z. (2021). Chinese medicine therapies for neurogenic bladder after spinal cord injury A protocol for systematic review and network meta-Analysis. Medicine (United States), 100(37). doi:10.1097/MD.0000000000027215	Research Protocol
Li Na. Meta-analysis of the effect of electroacupuncture combined with pelvic floor muscle exercise in the treatment of female stress urinary incontinence [J]. New Chinese Medicine, 2019, 51(08): 208-211. DOI: 10.13457/j.cnki.jncm.2019.08.062.	SRs/MAs of non-RCT
Fu Linhui, An Junming, Zhang Ding, Yang Pengcheng. Meta-analysis of electroacupuncture for neurogenic bladder after spinal cord injury [J]. Journal of Yunnan University of Traditional Chinese Medicine, 2019, 42(03): 61-68. DOI: 10.19288/j.cnki.issn.1000-2723.2019.03.011.	SRs/MAs of non-SUI
Liu Zhishun, Liu Baoyan, Yang Tao, Ye Yongming, Zhao Hong, Zhang Wei, Liu Jun, Liu Yuanshi, Guo Yufeng, Li Yisong, Huang Man, Yang Zhiqiang, Long Shuping, Huang Shixi. Clinical study of electroacupuncture in the treatment of senile urge urinary incontinence[1] J]. Chinese Acupuncture, 2001(10):5-8.	SRs/MAs of non-SUI
Tan Zhigao, Zhang Wei, Gong Houwu, Qin Zuoai, Zhong Feng, Cao Yue. Meta-analysis of the clinical efficacy of electroacupuncture in the treatment of post-stroke urinary incontinence [J]. Clinical Journal of Acupuncture and Moxibustion, 2015, 31(02): 74-77.	SRs/MAs of non-SUI
Wang Chaoran, Li Xiaojiang, Yang Peiying, Zhang Yao, Guo Shanqi, Jia Yingjie. Quality evaluation of literature reports on randomized controlled trials of acupuncture for postoperative urinary incontinence after prostate cancer [J]. Journal of Traditional Chinese Medicine Oncology, 2021, 3(04): 82-87.DOI:10.19811/j.cnki.ISSN2096-6628.2021.04.015.	SRs/MAs of non-SUI
Wang Jiaqi, Liu Zhishun, Yu Jinna, Zhang Wei. A systematic review on the treatment of neurogenic bladder dysfunction after spinal cord injury with acupuncture and moxibustion [J]. Henan Traditional Chinese Medicine, 2018, 38(03): 467-472. DOI: 10.16367/j.issn.1003-5028.2018.03.0124.	SRs/MAs of non-SUI
Wang Qiong, Cao Zhengliang, Sun Jiaqi, Li Saiqun, Zhou Youjun, Zhang Wei. A systematic review of the efficacy of acupuncture in the treatment of urge urinary incontinence [J]. Clinical Journal of Acupuncture and Moxibustion, 2015, 31(08): 50-52.	SRs/MAs of non-SUI
Wang Zailing, Fu Lixin, Xiong Jun, Qi Yingzhou, Li Sheng. A systematic review of the efficacy of acupuncture in the treatment of urinary incontinence after stroke [J]. Clinical Journal of Acupuncture and Moxibustion, 2010, 26(01): 39-43.	SRs/MAs of non-SUI
Xu Hairong, Liu Zhishun, Zhao Hong. A systematic review of acupuncture in the treatment of overactive bladder [J]. Journal of Modern Integrative Medicine, 2011, 20(04): 393-399.	SRs/MAs of non-SUI
Zhang Jiapeng, Chen Peiyi, Zhao Ziyu. Meta-analysis of clinical research on electroacupuncture for senile urinary incontinence [J]. Nursing Research, 2018, 32(07):1082-1087.	SRs/MAs of non-SUI
Guo Guangming, Yuan Baofeng, Zhu Shina, Li Jun. Meta-analysis of the efficacy of moxibustion combined with pelvic floor muscle training in the treatment of mild to moderate stress urinary incontinence [J]. Journal of Xiangnan University (Medical Edition), 2021, 23(03):13-18.DOI:10.16500/j.cnki.1673-498x.2021.03.003.	SRs/MAs of non-acupuncture
Liu Qinyu, Huang Huirong, Liu Fang, Han Xueqi, Miao Shaofang. Meta-analysis of the efficacy and quality of life of moxibustion on female stress urinary incontinence [J]. Massage and Rehabilitation Medicine, 2021, 12(04):8-14.DOI:10.19787/j.issn.1008-1879.2021.04.003.	SRs/MAs of non-acupuncture
Li Xiaoning, Yao Suyuan, Li Xiaowei, Ni Jinxia, Sheng Guobin. A clinical study of electroacupuncture on 120 cases of non-inhibitory neurogenic bladder [J]. Clinical Journal of Acupuncture and Moxibustion, 2005(05): 40-41.	Clinical Trials

**Table 3:** Characteristics of the included SRs/MAs.

Author, year (Country)	Trials (subjects)	Intervention group	Control group	Risk of bias assessment tool	Main results
Na Yang, 2021 (China) [18]	8 (607)	MA, EA, MA + control group, EA + control group	CM, SA, RT	Cochrane criteria	Based on this study, acupuncture intervention on SUI in middle-aged and elderly women can improve clinical efficacy, reduce urine leakage and decrease ICIQ-SF score in the urine pad test.
Xiuhua Lai, 2020 (China) [19]	15 (1,577)	EA	CM, SA, RT	Cochrane criteria	Electroacupuncture for women with SUI demonstrates significant efficacy and safety across key outcomes.
Yajing Zhong, 2020 (China) [20]	10 (1,200)	EA, EA + control group	CM, SA, RT	Cochrane criteria	In conclusion, our findings suggest that there is weak evidence for the use of EA to improve response rates, reduce urine leakage, and decrease incontinence episodes in patients with SUI.
Chen, et al. 2018 (China) [21]	14 (1,172)	EA, MA, MA + control group	RT, CM	Jadad scale	The acupuncture therapy was compared with other treatments, and the data analysis shows that the total effective rate of acupuncture in the treatment of female SUI is higher than that of the control group.
Chen, 2020 (China) [22]	11 (1,005)	EA, MA, MA + control group	RT, CM	Jadad scale	The clinical efficacy of acupuncture in the treatment of female SUI is significantly better than that of pelvic floor muscle exercises.
Ma, et al. 2021 (China) [23]	16 (985)	MA, EA, MA + control Group, EA + control Group	RT	Cochrane Criteria	Compared with Kegel exercise, acupuncture in the treatment of female SUI showed statistically significant differences in four commonly used indicators: Effective rate, ICI-Q-SF score, 1-hour urine pad test and 24-hour urine diary.
Wang, et al. 2014 (China) [24]	9 (579)	MA, EA, MA + control group, EA + control group	RT, CM, placebo	Jadad scale	The results show that acupuncture is effective in treating stress urinary incontinence, and is superior to western medicine and pelvic floor muscle training. It has no toxic side effects and is easy for patients to adhere to.
Zhang,et al. 2016 (China) [25]	10 (785)	EA, MA, EA + control group	RT, CM, placebo	Jadad scale	In conclusion, the analysis results show that the acupuncture prescription has some advantages in treating female SUI, but the limitations of inclusion in the study reduce the reliability of the above results.

**Table 4:** Result of the AMSTAR-2 assessments.

Author, year (Country)	Q1	**Q**2	Q3	**Q**4	Q5	Q6	**Q7**	Q8	**Q9**	Q10	**Q11**	Q12	**Q13**	Q14	**Q15**	Q16	Quality
Na Yang, 2021 (China) [18]	Y	PY	Y	PY	Y	Y	N	Y	Y	N	Y	Y	Y	N	Y	Y	VL
Xiuhua Lai, 2020 (China) [19]	Y	Y	Y	PY	Y	Y	N	Y	Y	N	Y	Y	Y	Y	Y	Y	VL
Yajing Zhong, 2020 (China) [20]	Y	Y	Y	PY	Y	Y	N	Y	Y	N	Y	Y	Y	Y	N	Y	VL
Chen, et al. 2018 (China) [21]	Y	PY	Y	PY	Y	Y	N	Y	Y	N	Y	Y	N	N	Y	N	VL
Chen, 2020 (China) [22]	Y	PY	Y	PY	Y	Y	N	Y	Y	N	Y	N	N	Y	N	N	VL
Ma, et al. 2021 (China) [23]	Y	PY	Y	PY	Y	N	N	Y	Y	N	Y	Y	Y	Y	Y	Y	VL
Wang, et al. 2014 (China) [24]	Y	PY	Y	PY	Y	N	N	Y	Y	N	Y	Y	Y	Y	Y	Y	VL
Zhang, and Xie 2016 (China) [25]	Y	PY	Y	PY	Y	N	N	Y	Y	N	Y	Y	Y	N	Y	N	VL

Note: Y, Yes; PY, partial Yes; N, No; VL, Very low; H, High. Note: Key areas are marked in bold.

**Table 5:** Results of the ROBIS assessments.

Author, year (Country)	Phase 1	Phase 2				Phase 3
	Assessing relevance	Domain 1: Study eligibility criteria	Domain 2: Identification and selection of studies	Domain 3: Collection and study appraisal	Domain 4: Synthesis and findings	Risk of bias in the review
Na Yang, 2021 (China) [18]	√	√	×	√	×	×
Xiuhua Lai, 2020 (China) [19]	√	√	×	√	√	×
Yajing Zhong, 2020 (China) [20]	√	√	×	√	×	×
Chen, et al. 2018 (China) [21]	√	√	×	√	×	×
Chen, 2020 (China) [22]	√	√	×	√	×	×
Ma, et al. 2021 (China) [23]	√	√	×	×	√	×
Wang, et al. 2014 (China) [24]	√	√	×	×	×	×
Zhang and Xie, 2016 (China) [25]	√	√	×	√	×	×

Note:√, low risk; ×, high risk.

**Table 6:** Results of the PRISMA checklist.

Section/topic		Items	Na Yang, 2021 (China) [18]	Xiuhua Lai, 2020 (China) [19]	Yajing Zhong, 2020 (China) [20]	Chen, et al. 2018 (China) [21]	Chen, 2020 (China) [22]	Ma, et al. 2021 (China) [23]	Wang, et al. 2014 (China) [24]	Zhang and Xie 2016 (China) [25]	Number of yes or partially yes (%)
Title	Title	Item 1	Y	Y	Y	Y	Y	Y	Y	Y	100%
Abstract	Abstract	Item 2	PY	PY	PY	PY	PY	PY	PY	PY	100%
Introduction	Rationale	Item 3	Y	Y	Y	Y	Y	Y	Y	Y	100%
	Objectives	Item 4	Y	Y	Y	Y	Y	Y	Y	Y	100%
Methods	Eligibility criteria	Item 5	Y	Y	Y	Y	Y	Y	Y	Y	100%
	Information sources	Item 6	Y	Y	Y	Y	Y	Y	Y	Y	100%
	Search strategy	Item 7	N	N	N	N	N	N	N	N	0%
	Selection process	Item 8	Y	Y	Y	Y	Y	Y	Y	Y	100%
	Data collection process	Item 9	Y	Y	Y	Y	Y	N	N	N	62.50%
	Data items	Item 10 (a)	Y	Y	Y	Y	Y	Y	Y	Y	100%
		Item 10 (b)	PY	PY	PY	PY	PY	PY	PY	PY	100%
	Study risk of bias assessment	Item 11	Y	Y	Y	Y	Y	PY	PY	Y	100%
	Effect measures	Item 12	Y	Y	Y	Y	Y	Y	Y	Y	100%
	Synthesis methods	Item 13 (a)	Y	Y	Y	Y	Y	Y	Y	Y	100%
		Item 13 (a)	Y	Y	Y	Y	Y	Y	Y	Y	100%
		Item 13 (c)	Y	Y	Y	Y	Y	Y	Y	Y	100%
		Item 13 (d)	Y	Y	Y	Y	Y	Y	Y	Y	100%
		Item 13 (e)	N	Y	Y	N	Y	Y	Y	N	62.50%
		Item 13 (f)	N	Y	Y	N	Y	Y	N	Y	62.50%
	Reporting bias assessment	Item 14	Y	Y	N	Y	N	Y	N	Y	62.50%
	Certainty assessment	Item 15	N	N	N	N	N	N	N	N	0%
Results	Study selection	Item 16 (a)	Y	Y	Y	Y	Y	Y	Y	Y	100%
		Item 16 (b)	Y	Y	Y	Y	Y	N	Y	Y	87.50%
	Study characteristics	Item 17	Y	Y	Y	Y	Y	Y	Y	Y	100%
	Risk of bias in studies	Item 18	Y	Y	Y	Y	Y	Y	Y	Y	100%
	Results of individual studies	Item 19 (a)	Y	Y	Y	Y	Y	Y	Y	Y	100%
		Item 19 (b)	Y	Y	Y	Y	Y	Y	Y	Y	100%
	Results of syntheses	Item 20 (a)	Y	Y	Y	Y	Y	Y	Y	Y	100%
		Item 20 (b)	Y	Y	Y	Y	Y	Y	Y	Y	100%
		Item 20 (c)	N	Y	Y	N	Y	Y	Y	N	62.50%
		Item 20 (d)	N	Y	Y	N	Y	Y	N	Y	62.50%
	Reporting biases	Item 21	Y	Y	N	Y	N	Y	Y	Y	75%
	Certainty of evidence	Item 22	N	N	N	N	N	N	N	N	0%
Discussion	Discussion	Item 23 (a)	Y	Y	Y	Y	Y	Y	Y	Y	100%
		Item 23 (b)	Y	Y	Y	Y	Y	Y	Y	Y	100%
		Item 23 (c)	Y	Y	Y	Y	Y	Y	Y	Y	100%
		Item 23 (d)	Y	Y	Y	Y	Y	Y	Y	Y	100%
Other information	Registration and protocol	Item 24 (a)	N	Y	Y	N	N	N	N	N	25%
		Item 24 (b)	N	Y	Y	N	N	N	N	N	25%
		Item 24 (c)	N	N	N	N	N	N	N	N	0%
	Support	Item 25	Y	Y	Y	N	N	Y	Y	N	62.50%
	Competing interests	Item 26	Y	Y	Y	N	N	N	N	N	37.50%
	Availability of data, code, and other materials	Item 27	Y	Y	Y	Y	Y	Y	Y	Y	100%

Note: Y, yes; N, no; PY, partially yes.

**Table 7:** Results of certainty of quality.

Author, year (Country)	Outcomes	Studies (participants)	Limitations	Inconsistency	Indirectness	Imprecision	Publication bias	Quality
Na Yang, 2021 (China) [18]	Effective rate	7 (523)	0	0	0	0	0	High
	1-hour pad test	5 (417)	0	−1②	0	0	−1④	Low
	ICIQ-SF score	4 (366)	0	−1②	0	−1③	−1④⑤	Low
Xiuhua Lai, 2020 (China) [19]	Effective rate	13 (1,333)	0	0	0	0	−1④	Moderate
	ICIQ-SF score	6 (763)	0	−1②	0	0	−1④	Low
	1-hour pad test	5 (900)	0	−1②	0	0	−1④	Low
Yajing Zhong, 2020 (China) [20]	Effective rate	7 (1,010)	0	−1②	0	0	−1④	Low
	1-hour pad test	9 (1,157)	0	−1②	0	0	−1④	Low
	ICIQ-SF score	9 (1,157)	0	−1②	0	0	−1④	Moderate
	72-hour incontinence episodes	3 (654)	0	0	0	0	−1④	Moderate
	Follow-up of the effective rate	2 (584)	0	0	0	0	−1④⑤	Moderate
	Follow-up of the ICIQ-SF score	3 (644)	0	−1②	0	0	−1④⑤	Moderate
	Follow-up of the 72-hour incontinence episodes	2 (584)	0	0	0	0	−1④⑤	Moderate
Chen, et al. 2018 (China) [21]	Effective rate (acupuncture and RT)	8 (558)	−1①	0	0	0	0	Moderate
	Effective rate (acupuncture and CM)	3 (220)	−1①	0	0	−1③	−1④	Low
	ICIQ-SF score (acupuncture and RT)	5 (323)	−1①	−1②	0	−1③	−1④	Very low
Chen, 2020 (China) [22]	Effective rate	7 (577)	−1①	0	0	0	−1④	Low
Ma, et al. 2021 (China) [23]	Effective rate	13 (812)	−1①	0	0	0	−1④	Low
	ICIQ-SF score	6 (377)	−1①	−1②	0	−1③	−1④	Very low
	1-hour pad test	9 (504)	−1①	−1②	0	0	−1④	Very low
	24-hour urination diary	9 (143)	−1①	0	0	−1③	−1④	Low
Wang, et al. 2014 (China) [24]	Effective rate (acupuncture and RT)	5 (461)	−1①	0	0	0	0	Moderate
	Effective rate (acupuncture and CM)	3 (220)	−1①	0	0	−1③	−1⑤	Moderate
	Effective rate (acupuncture and placebo)	2 (198)	−1①	0	0	−1③	−1⑤	Moderate
Zhang and Xie, 2016 (China) [25]	Effective rate	10 (607)	−1①	−1②	0	0	−1④	Low
	ICIQ-SF score	4 (257)	−1①	−1②	0	−1③	−1④	Very low
	VAS	2 (206)	0	0	0	−1③	−1④⑤	Moderate

Note: ① The included studies have a large bias in methodology such as randomization, allocation concealment, and blinding. ② The confidence interval overlaps less or the I2 value of the combined results was larger. ③ The sample size from the included studies does not meet the optimal sample size or the 95% confidence interval crosses the invalid line. ④ The funnel chart is asymmetry. ⑤ Fewer studies were included, and their results were all positive, which may result in a large publication bias.

**Table 8:** Summary of evidence.

Author, year (country)	Outcomes	Studies (participants, intervention group/control group)	Relative effect (95% CI)	Heterogeneity	*p* value
Na Yang, 2021 (China) [18]	Effective rate	7 (523, 264/259)	OR = 5.52 (3.13, 9.73)^*∗*^	*I* ^2^ = 0%	*p* < 0.00001
	1-hour pad test	5 (417, 210/207)	SMD = −2.67 (−4.05, −1.29)^*∗*^	*I* ^2^ = 96%	*p*=0.0001
	ICIQ-SF score	4 (366, 183/183)	MD = −3.46, (−3.69, −3.22)^*∗*^	*I* ^2^ = 87%	*p* < 0.00001
Xiuhua Lai, 2020 (China) [19]	Effective rate	13 (1,333, 667/666)	OR = 5.64 (4.19, 7.59)^*∗*^	*I* ^2^ = 22%	*p* < 0.00001
	ICIQ-SF score	6 (763, 381/382)	SMD = −0.61 (−0.74, −0.48)^*∗*^	*I* ^2^ = 80%	*p* < 0.00001
	1-hour pad test	5 (900, 450/450)	MD = −4.14 (−4.96, −3.33)^*∗*^	*I* ^2^ = 78%	*p* < 0.00001
Yajing Zhong, 2020 (China) [20]	Effective rate	7 (1,010, 503/507)	RR = 2.03 (1.40, 2.95)^*∗*^	*I* ^2^ = 89%	*p*=0.0002
	1-hour pad test	9 (1,157, 578/579)	MD = 3.33 (0.89, 5.77)^*∗*^	*I* ^2^ = 98%	*p*=0.008
	ICIQ-SF score	9 (1,157, 578/579)	MD = 3.14 (2.42, 3.85)^*∗*^	*I* ^2^ = 63%	*p* < 0.00001
	72-hour incontinence episodes	3 (654, 327/327)	MD = 1.17 (0.56, 1.78)^*∗*^	*I* ^2^ = 0%	*p*=0.0002
	Follow-up of the effective rate	2 (584, 292/292)	MD = 2.10 (1.28, 2.92)^*∗*^	*I* ^2^ = 0%	*p* < 0.00001
	Follow-up of the ICIQ-SF score	3 (644, 322/322)	MD = 2.89 (1.96, 3.82)^*∗*^	*I* ^2^ = 54%	*p* < 0.00001
	Follow-up of the 72-hour incontinence episodes	2 (584, 292/292)	MD = 2.10 (1.28, 2.92)^*∗*^	*I* ^2^ = 0%	*p* < 0.00001
Chen, et al. 2018 (China) [21]	Effective rate (acupuncture and RT)	8 (558, 281/277)	RR = 1.33 (1.22, 1.46)^*∗*^	*I* ^2^ = 0%	*p* < 0.00001
	Effective rate (acupuncture and CM)	3 (220, 110/110)	RR = 2.15 (1.64, 2.83)^*∗*^	*I* ^2^ = 0%	*p* < 0.00001
	ICIQ-SF score (acupuncture and RT)	5 (323, 162/161)	MD = −1.29 (−2.88, 0.31)	*I* ^2^ = 80%	*p*=0.11
Chen, 2020 (China) [22]	Effective rate	7 (577, 289/287)	OR = 4.10 (1.85, 9.10)^*∗*^	*I* ^2^ = 62%	*p*=0.0005
Ma, et al. 2021 (China) [23]	Effective rate	13 (812, 408/404)	OR = 6.04 (3.84, 9.49)^*∗*^	*I* ^2^ = 0%	*p* < 0.00001
	ICIQ-SF score	6 (377, 189/188)	MD = −3.03 (−4.17, −1.90)^*∗*^	*I* ^2^ = 80%	*p* < 0.00001
	1-hour pad test	9 (504, 252/252)	MD = −2.95 (−3.86, −2.04)^*∗*^	*I* ^2^ = 88%	*p* < 0.00001
	24-hour urination diary	9 (143, 71/72)	MD = −0.97 (−1.61, −0.33)^*∗*^	*I* ^2^ = 65%	*p* < 0.00001
Wang, et al. 2014 (China) [24]	Effective rate (acupuncture and RT)	5 (461, 231/230)	OR = 4.00 (2.51, 6.39)^*∗*^	*I* ^2^ = 0%	*p*=0.003
	Effective rate (acupuncture and CM)	3 (220, 110/110)	OR = 9.14 (4.77, 17.53)^*∗*^	*I* ^2^ = 47%	*p* < 0.00001
	Effective rate (acupuncture and placebo)	2 (198, 99/99)	OR = 3.05 (1.59, 5.84)^*∗*^	*I* ^2^ = 0%	*p*=0.0008
Zhang and Xie 2016 (China) [25]	Effective rate	10 (785, 394/391)	OR = 4.27 (2.42, 7.56)^*∗*^	*I* ^2^ = 50%	*p* < 0.00001
	ICIQ-SF score	4 (257, 129/128)	SMD = −0.41 (−1.00, 0.18)	*I* ^2^ = 82%	*p*=0.17
	VAS	2 (206, 103/103)	SMD = −2.16 (−2.51, −1.81)^*∗*^	*I* ^2^ = 0%	*p* < 0.00001

Note: ^*∗*^ The 95% confidence interval does not cross the invalid line.

## Data Availability

The datasets analyzed during the current study are available from the corresponding author upon reasonable request.

## Supplementary Materials

Supplementary Materials Supplementary file 1: List of articles excluded from this study.
