# Supplementary material for: A Critical Overview of Systematic Reviews and Meta-Analyses of Acupuncture for Female Stress Urinary Incontinence
Source: Pain Res Manag. 2022 May 17;2022:5887862. doi: 10.1155/2022/5887862 (PMC9129990; doi:10.1155/2022/5887862)
Supplement: Supplementary Materials — Supplementary file 1: List of articles excluded from this study. [file 5887862.f1.docx]

| **Report excluded** | **Reason for Exclusion** |
| --- | --- |
| Cheng, P., Chi, Z., Xiao, Y., Xie, W., Zhu, D., Yu, T., . . . Jiao, L. (2020). The acupuncture-related therapy for post-stroke urinary incontinence: A protocol for systematic review and network meta-analysis. Medicine (Baltimore), 99(44), e22865. doi:10.1097/md.0000000000022865 | Research Protocol |
| Huang, W., Li, X., Wang, Y., Yan, X., & Wu, S. (2017). Electroacupuncture for women with stress urinary incontinence: Protocol for a systematic review and meta-analysis. Medicine (Baltimore), 96(49), e9110. doi:10.1097/md.0000000000009110 | Research Protocol |
| Lin, Q., Ren, Y., Chen, K., Duan, H., Chen, M., & Liu, C. (2021). Efficacy and safety of heat-sensitive moxibustion in the treatment of neurogenic bladder after spinal cord injury: A protocol for systematic review and meta-analysis. Medicine (Baltimore), 100(24), e26424. doi:10.1097/md.0000000000026424 | Research Protocol |
| Mo, Q., Wang, Y., Ye, Y., Yu, J., & Liu, Z. (2015). Acupuncture for adults with overactive bladder: a systematic review protocol. BMJ Open, 5(1), e006756. doi:10.1136/bmjopen-2014-006756 | Research Protocol |
| Su, T., Zhou, J., Liu, Z., Chen, Y., Zhang, W., Chu, H., . . . Liu, B. (2015). The efficacy of electroacupuncture for the treatment of simple female stress urinary incontinence - comparison with pelvic floor muscle training: study protocol for a multicenter randomized controlled trial. Trials, 16, 45. doi:10.1186/s13063-015-0560-1 | Research Protocol |
| Sun, Z., Yu, N., Yue, J., & Zhang, Q. (2016). Acupuncture for urinary incontinence after stroke: a protocol for systematic review. BMJ Open, 6(2), e008062. doi:10.1136/bmjopen-2015-008062 | Research Protocol |
| Wang, P., Shi, J., Zhao, L., Li, M., Jiao, J., Li, L., . . . Zhang, S. (2020). The efficacy and safety of electroacupuncture against urinary incontinence after stroke: A protocol for systematic review and meta analysis. Medicine (Baltimore), 99(38), e22275. doi:10.1097/md.0000000000022275 | Research Protocol |
| Wang, T. S., Wang, Z. M., Zhao, Y., Tang, Z. C., Song, W. D., & Wang, G. K. (2020). Effectiveness of electroacupuncture (EA) for the treatment of urinary incontinence (UI) in patients with spinal cord injury (SCI): A protocol of systematic review of randomized controlled trials. Medicine (Baltimore), 99(30), e21077. doi:10.1097/md.0000000000021077 | Research Protocol |
| Wang, Y., Li, H., Wang, J., Hao, Q., Tu, Y., Chen, Y., . . . Zhu, T. (2020). A network meta-analysis protocol of conservative interventions for urinary incontinence in postpartum women. Medicine (Baltimore), 99(33), e21772. doi:10.1097/md.0000000000021772 | Research Protocol |
| Yang, J., Cheng, Y., Zhao, L., Chen, J., Zheng, Q., Guo, Y., & Liang, F. (2020). Acupuncture and related therapies for stress urinary incontinence: A protocol for systematic review and network meta-analysis. Medicine (Baltimore), 99(28), e21033. doi:10.1097/md.0000000000021033 | Research Protocol |
| Zhong, D., Tang, W., Geng, D., & He, C. (2019). Efficacy and safety of acupuncture therapy for urinary incontinence in women: A systematic review and meta- analysis. Medicine (Baltimore), 98(40), e17320. doi:10.1097/md.0000000000017320 | Research Protocol |
| Zhu, Z., Zhuo, Y., Jin, H., Wu, B., & Li, Z. (2021). Chinese medicine therapies for neurogenic bladder after spinal cord injury A protocol for systematic review and network meta-Analysis. Medicine (United States), 100(37). doi:10.1097/MD.0000000000027215 | Research Protocol |
| Li Na. Meta-analysis of the effect of electroacupuncture combined with pelvic floor muscle exercise in the treatment of female stress urinary incontinence [J]. New Chinese Medicine, 2019, 51(08): 208-211. DOI: 10.13457/j.cnki.jncm.2019.08 .062. | SRs/MAs of non-RCT |
| Fu Linhui, An Junming, Zhang Ding, Yang Pengcheng. Meta-analysis of electroacupuncture for neurogenic bladder after spinal cord injury [J]. Journal of Yunnan University of Traditional Chinese Medicine, 2019, 42(03): 61-68. DOI: 10.19288 /j.cnki.issn.1000-2723.2019.03.011. | SRs/MAs of non-SUI |
| Liu Zhishun, Liu Baoyan, Yang Tao, Ye Yongming, Zhao Hong, Zhang Wei, Liu Jun, Liu Yuanshi, Guo Yufeng, Li Yisong, Huang Man, Yang Zhiqiang, Long Shuping, Huang Shixi. Clinical study of electroacupuncture in the treatment of senile urge urinary incontinence[1] J]. Chinese Acupuncture, 2001(10):5-8. | SRs/MAs of non-SUI |
| Tan Zhigao, Zhang Wei, Gong Houwu, Qin Zuoai, Zhong Feng, Cao Yue. Meta-analysis of the clinical efficacy of electroacupuncture in the treatment of post-stroke urinary incontinence [J]. Clinical Journal of Acupuncture and Moxibustion, 2015, 31(02): 74-77. | SRs/MAs of non-SUI |
| Wang Chaoran, Li Xiaojiang, Yang Peiying, Zhang Yao, Guo Shanqi, Jia Yingjie. Quality evaluation of literature reports on randomized controlled trials of acupuncture for postoperative urinary incontinence after prostate cancer [J]. Journal of Traditional Chinese Medicine Oncology, 2021, 3( 04):82-87.DOI:10.19811/j.cnki.ISSN2096-6628.2021.04.015. | SRs/MAs of non-SUI |
| Wang Jiaqi, Liu Zhishun, Yu Jinna, Zhang Wei. A systematic review on the treatment of neurogenic bladder dysfunction after spinal cord injury with acupuncture and moxibustion [J]. Henan Traditional Chinese Medicine, 2018, 38(03): 467-472. DOI: 10.16367/ j.issn.1003-5028.2018.03.0124. | SRs/MAs of non-SUI |
| Wang Qiong, Cao Zhengliang, Sun Jiaqi, Li Saiqun, Zhou Youjun, Zhang Wei. A systematic review of the efficacy of acupuncture in the treatment of urge urinary incontinence [J]. Clinical Journal of Acupuncture and Moxibustion, 2015, 31(08): 50-52. | SRs/MAs of non-SUI |
| Wang Zailing, Fu Lixin, Xiong Jun, Qi Yingzhou, Li Sheng. A systematic review of the efficacy of acupuncture in the treatment of urinary incontinence after stroke [J]. Clinical Journal of Acupuncture and Moxibustion, 2010, 26(01): 39-43. | SRs/MAs of non-SUI |
| Xu Hairong, Liu Zhishun, Zhao Hong. A systematic review of acupuncture in the treatment of overactive bladder [J]. Journal of Modern Integrative Medicine, 2011, 20(04): 393-399. | SRs/MAs of non-SUI |
| Zhang Jiapeng, Chen Peiyi, Zhao Ziyu. Meta-analysis of clinical research on electroacupuncture for senile urinary incontinence [J]. Nursing Research, 2018,32(07):1082-1087. | SRs/MAs of non-SUI |
| Guo Guangming, Yuan Baofeng, Zhu Shina, Li Jun. Meta-analysis of the efficacy of moxibustion combined with pelvic floor muscle training in the treatment of mild to moderate stress urinary incontinence [J]. Journal of Xiangnan University (Medical Edition), 2021,23(03 ):13-18.DOI:10.16500/j.cnki.1673-498x.2021.03.003. | SRs/MAs of non-acupuncture |
| Liu Qinyu, Huang Huirong, Liu Fang, Han Xueqi, Miao Shaofang. Meta-analysis of the efficacy and quality of life of moxibustion on female stress urinary incontinence [J]. Massage and Rehabilitation Medicine, 2021,12(04):8-14.DOI :10.19787/j.issn.1008-1879.2021.04.003. | SRs/MAs of non-acupuncture |
| Li Xiaoning, Yao Suyuan, Li Xiaowei, Ni Jinxia, Sheng Guobin. A clinical study of electroacupuncture on 120 cases of non-inhibitory neurogenic bladder [J]. Clinical Journal of Acupuncture and Moxibustion, 2005(05): 40-41. | Clinical Trials |
